# Analysis of the Virus Dynamics Model Reveals That Early Treatment of HCV Infection May Lead to the Sustained Virological Response

**DOI:** 10.1371/journal.pone.0041209

**Published:** 2012-07-24

**Authors:** Saurabh Gupta, Raghvendra Singh

**Affiliations:** Department of Chemical Engineering, Indian Institute of Technology Kanpur, Kanpur, India; University of Modena & Reggio Emilia, Italy

## Abstract

Considerable progress has been made towards understanding hepatitis C virus, its pathogenesis and the effect of the drug therapy on the viral load, yet around 50% of patients do not achieve the sustained virological response (SVR) by the standard treatment. Although several personalized factors such as patients’ age and weight may be important, by mathematical modeling we show that the time of the start of the therapy is a significant factor in determining the outcome. Toward this end, we first performed sensitivity analysis on the standard virus dynamics model. The analysis revealed four phases when the sensitivity of the infection to drug treatment differs. Further, we added a perturbation term in the model to simulate the drug treatment period and predict the outcome when the therapy is carried out during each of the four phases. The study shows that while the infection may be difficult to treat in the late phases, the therapy is likely to result in SVR if it is carried out in the first or second phase. Thus, development of newer and more sensitive screening methods is needed for the early detection of the infection. Moreover, the analysis predicts that the drug that blocks new infections is more effective than the drug that blocks the virus production.

## Introduction

Hepatitis C, a single stranded RNA virus belonging to flaviviridae family, has a high prevalence rate with an estimated 170 million likely carrier worldwide [1]. Around 4.1 million individuals may be carrying the virus in the U.S. alone, majority developing the chronic infection [2]. Chronic infection may progressively cause liver fibrosis, resulting in cirrhosis in around 20% of the patients [1], [3]. It has also been linked to development of hepatocellular carcinoma [4], with likely role of HCV core protein [5]. The antiviral cytokine, interferon-α, had been the corner stone of the chronic HCV treatment for many years. However, treatment with IFN-α was effective in achieving the sustained virological response (SVR) in less than 20% of the patients [6]. The combination therapy, which includes ribavirin along with IFN-a, and replacement of IFN-α by pegylated interferon have further improved the treatment, increasing the SVR rate to more than 50% [6], [7], [8]. More recently, a HCV protease inhibitor, telaprevir, has shown potential to improve the SVR rate further [9], [10], an inhibitor of HCV NS5A, a protein critical in viral life cycle and a likely drug target, has been identified [11], and many more drugs are undergoing clinical trials. Thus, considerable progress has been made in treating the infection and identifying newer targets for the drug therapy. Yet, in a significant percentage of the patients, the infection persists or resurges after the completion of the treatment, many developing liver cirrhosis and cancer. Therefore, better understanding of the role of the drugs in achieving SVR as well as the progression of disease is needed.

A model of virus dynamics has been previously described [12]–[16]. It has helped in explaining multiple aspects of HIV [17], [18], [19], HBV [14], and HCV [20], [21] infections. The in-vivo study on the effect of the IFN-α showed a biphasic response and it was found that IFN-α likely decreases the initial viral load mainly by blocking the virus production from the infected cells [20]. In some patients, besides the two phases of decline of the viral load, an intermediate shoulder phase, in which the viral load remains nearly constant [22]–[25], has been observed. This triphasic viral load decay has been explained by taking into account the proliferation of hepatocytes [25], [26], [27] in the original model. Besides explaining many observed viral decay profiles, the model also revealed that for the efficacies of the drug higher than a critical value, the infection will be cleared during the treatment and for efficacies lower than the critical value, a new steady state of infection may be reached [26], [27]. Thus, the likelihood of achieving SVR as well as development of drug resistance may depend on the efficacy of the drug treatment [18].

Although a lot is known about IFN-α, its role in modulating immune response, and its antiviral activity, it is not clear why the therapy fails to achieve SVR in around 50% of cases. The response of the therapy may depend on factors such as: the HCV genotype [28]–[31], level of hepatic fibrosis [32], [33], the viral load [29], body weight and age of the patient [34], [35]. Besides these variables, it is known that replacing IFN-α with pegylated interferon increases the chances of achieving SVR significantly, likely due to better half life and bioavailability of the drug, and a higher dose of pegylated interferon is more effective in achieving SVR than a lower dose [8]. Moreover, a significant percentage of the patients, who were previously treated with IFN-α and ribavirin combination therapy, achieved SVR when retreated with pegylated interferon and ribavirin [36]. Furthermore, retreating the patients with higher dose of IFN-α for 6 months caused 29% of the patients to achieve SVR [37] and it has been found that interferon alfa-2b decreases viral load in a dose dependent manner [38]. Thus, the dose of the drug and its bioavailability may be an important factor in determining the outcome of the therapy [38] and regression analysis based on clinical data has been used to predict the dose of IFN plus ribavirin that may be required to maximize the number of patients who clear the virus during the therapy [39].

These studies prompted us to look mechanistically into the role of effective dosing of the drug in achieving SVR. We performed the sensitivity analysis on the standard virus dynamics model. The analysis revealed that there are four time periods, in which the sensitivity of the infection to drug treatment varies. Based on this finding, we added a perturbation term in the model to simulate the drug treatment during a specific phase of the infection. The perturbation analysis showed that the first and second phases are the most effective for the antiviral therapy. Further, the study shows that the drug that blocks new infections is more potent in achieving SVR than the drug that blocks the virus production.

**Figure 1:**
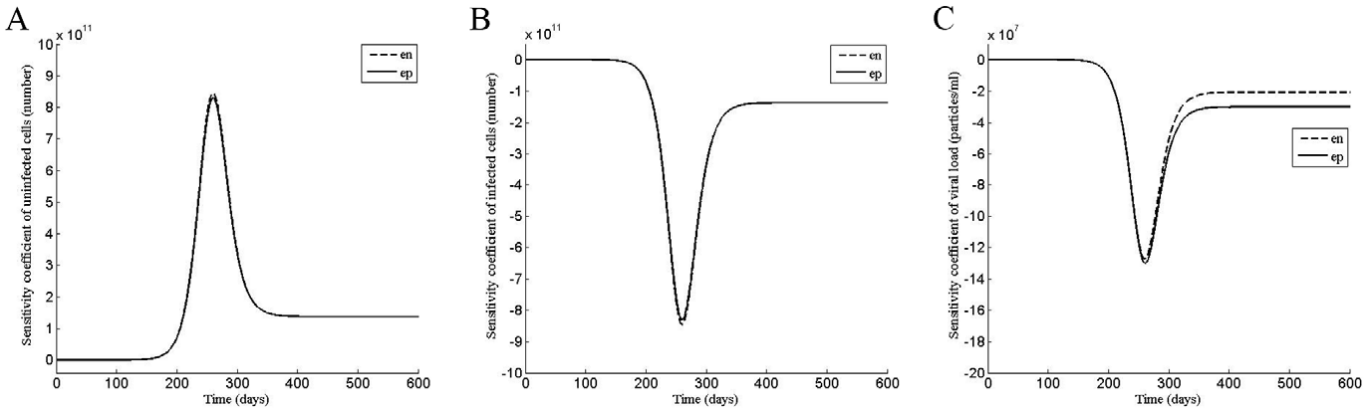
Sensitivity analysis of the model shows different phases of effectiveness of the drug. Sensitivity coefficient of the (A) uninfected cells, (B) infected cells, and (C) the viral load with respect to the efficacies, ε_n_ (e_n_) and ε_p_ (e_p_) were plotted as a function of time for ε_n_ = 0 and ε_p_ = 0.

## Methods

### Model Formulation

We started with the standard model [12]–[16] of the virus dynamics and considered two possible modes of action of the drug, which are: (i) to block new infections (ii) to block virus production from the productively infected cells.
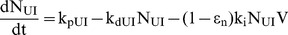
(1)

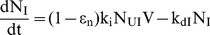
(2)

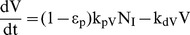
(3)where,

N_UI_: Number of uninfected hepatocytes; N_I_: Number of infected hepatocytes; V: Viral load (particles/ml); k_pUI_ : Production rate of the uninfected hepatocytes (number of cells/day); k_dUI_: Death rate constant of the uninfected hepatocytes (per day); k_i_: Infection rate constant (ml/day/virus particle); k_dI_: Death rate constant of the infected cells (per day); k_pV_: Production rate constant of the virus (particles/ml/day/infected cell); k_dV_: Virus clearance rate constant (per day); ε_n_: Efficacy of the drug in blocking new infections; ε_p_: Efficacy of the drug in blocking the production of the virus from the infected cells.

Next, we performed the sensitivity analysis on the model with respect to the two efficacy parameters and found the sensitivity coefficients of the uninfected (S_UI1_), infected (S_I1_) cells, and the viral load (S_V1_) with respect to ε_n_, and the sensitivity coefficients S_UI2_, S_I2_, S_V2_ of the three variables with respect to ε_p_. The sensitivity coefficient vs time plots for ε_n_ = 0 and, ε_p_ = 0 were fitted in polynomials using nonlinear regression to get each sensitivity coefficient as a function of time:

S_UI1_ =  f_UI1_ (t); S_I1_ =  f_I1_ (t); S_V1_ =  f_V1_(t); S_UI2_ =  f_UI2_ (t); S_I2_ =  f_I2_ (t); and S_V2_ =  f_V2_(t).

The equations (1), (2), and (3) for ε_n_ = 0 and ε_p_ = 0 will achieve a steady state, which represents the infection state without drug treatment.

**Figure 2:**
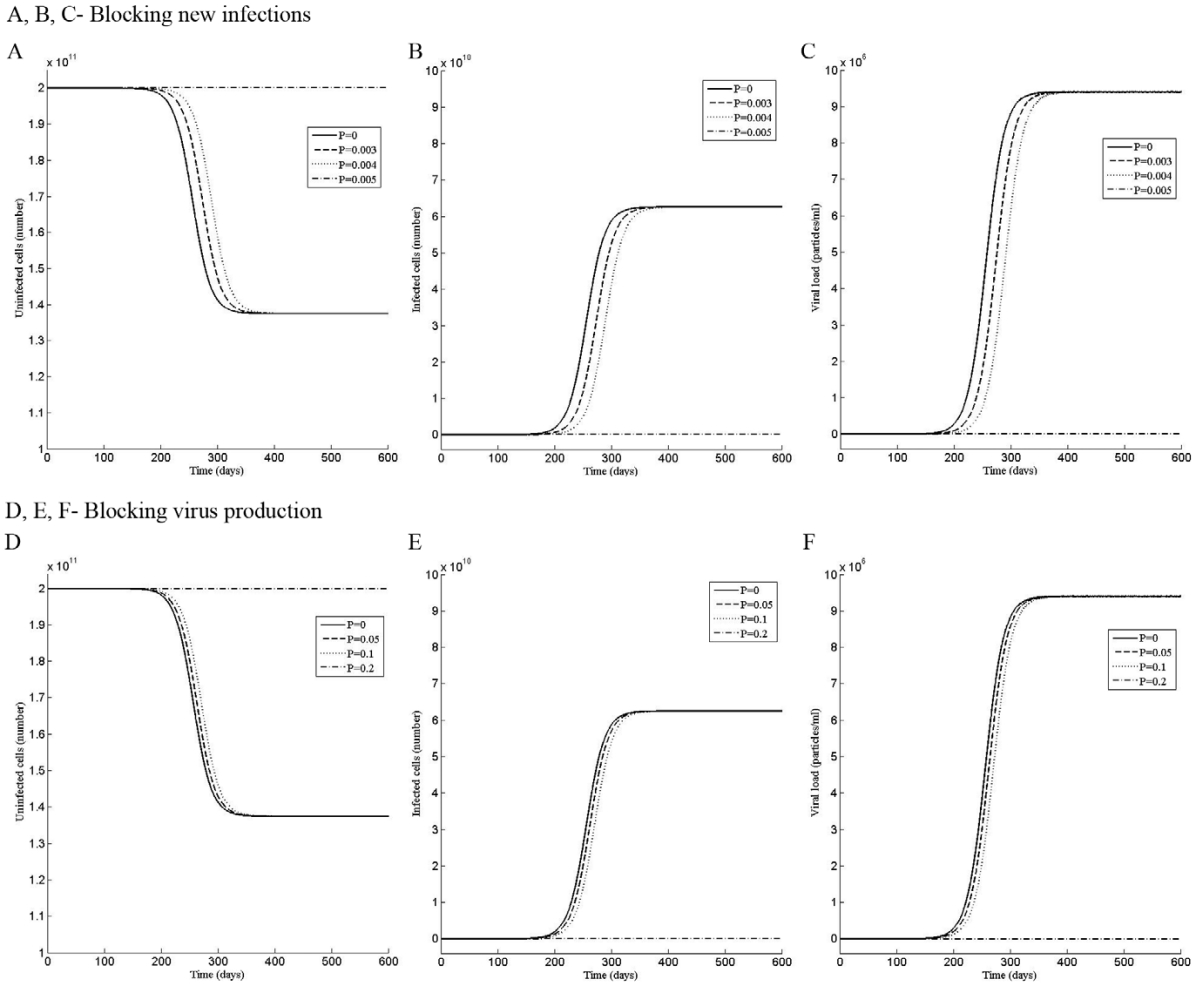
Perturbation in the first phase shows two steady states of the infection. Dynamics of the (A) uninfected cells, (B) infected cells, and (C) the viral load when the system was perturbed by the dose P of the drug that blocks new infections during the time period t = 0 to t = 180 day. Dynamics of the (D) uninfected cells, (E) infected cells, and (F) the viral load when the system was perturbed by the dose P of the drug that blocks virus production during the time period t = 0 to t = 180 day.

To find the steady state when a drug, which blocks *de novo* infections, of dose S is administered starting at any time t = t_1_ for a period Δt ( = 6 months), we added a perturbation term in the equation (2) using Taylor series approximation. Thus, during the Δt period, the corresponding equations for the uninfected, infected cells and the viral load can be written as:
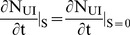
(4)

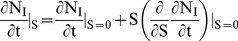
(5)

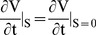
(6)


The efficacy is a saturating function of the dose of the drug. Thus,
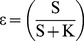
(7)where, K is the concentration of the drug needed to give 50% efficacy.

Therefore, equation (5) can be written as:
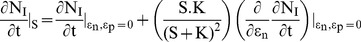
(8)or,
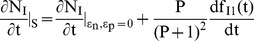
(9)where, 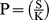 is the normalized dose of the drug.

Thus, for the drug, whose mode of the action is to block *de novo* infection, the governing equations can be written as:
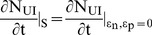
(10)

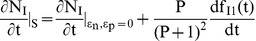
(11)

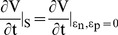
(12)


Similarly, for the drug, which blocks the virus production, the equations for the dynamics of the uninfected, infected cells and the viral load can be written as:
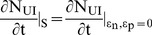
(13)

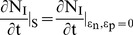
(14)

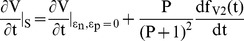
(15)


The equations (1), (2), and (3) with ε_n_ = 0 and ε_p_ = 0 for all times during infection, except when the therapy is being carried out, have been combined with the equations (10), (11), and (12) or with (13), (14), and (15), applicable for the Δt period for respective mode of action of the drug. In the study, we have not included the pharmacokinetics of the drugs and have assumed that the drug concentration remains constant during the duration of the therapy.

**Figure 3:**
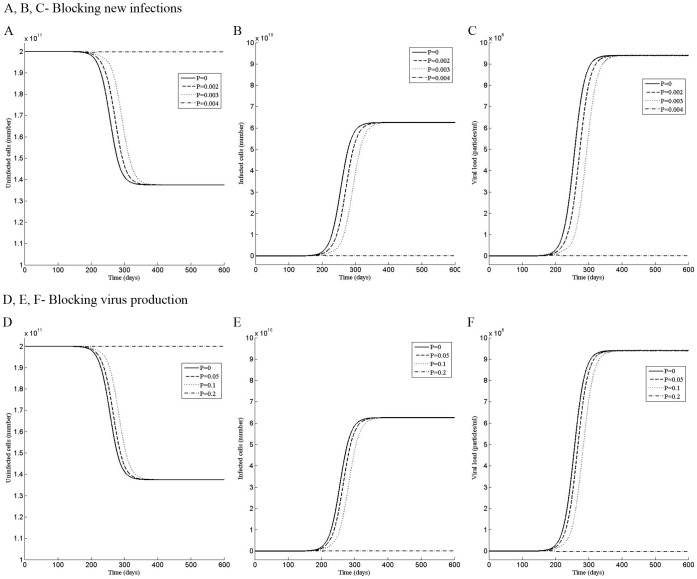
Perturbation in the second phase shows two steady states of the infection. Dynamics of the (A) uninfected cells, (B) infected cells, and (C) the viral load when the system was perturbed by the dose P of the drug that blocks new infections during the time period t = 80 to t = 260 day. Dynamics of the (D) uninfected cells, (E) infected cells, and (F) the viral load when the system was perturbed by the dose P of the drug that blocks virus production during the time period t = 80 to t = 260 day.

### Rate Constants

k_dV_ = 6.2 day^−1^; k_dI_ = 0.14 day^−1^ [20]; k_pUI_ = 2.8×10^10^ number of cells/day. Death rate constant of the uninfected hepatocytes (k_dUI_) has been assumed to be the same as that of the infected hepatocytes. Average number of HCV particles produced per infected cell in its life time is 33.28 [40] and the average plasma volume in human is taken as 5000 ml. Therefore, the production rate constant of the virus (k_pV_) has been calculated as 0.00093184 particles/ml/day/infected cells. The mean value of the viral load is taken as 9.4×10^6^ [20]. The infection rate constant (k_i_) has been calculated for the mean viral load and found to be 6.7766×10^−9^ ml/day/virus particle.

### Initial Conditions

At time t = 0, the number of uninfected hepatocytes are 2×10^11^ [20] and infected hepatocytes are zero. It is assumed that a person is initially infected with 0.2 ml of fluid containing 1×10^6^ virus particles/ml. Therefore, the initial viral load has been calculated as 40 particles/ml.

### Computations

Equations 1–15 were solved using ODE solver ode45 of MATLAB version 7.7.0 (R2008b). The sensitivity coefficient vs time plots were fitted in polynomials using nonlinear regression program of Microsoft Office Excel 2003.

**Figure 4:**
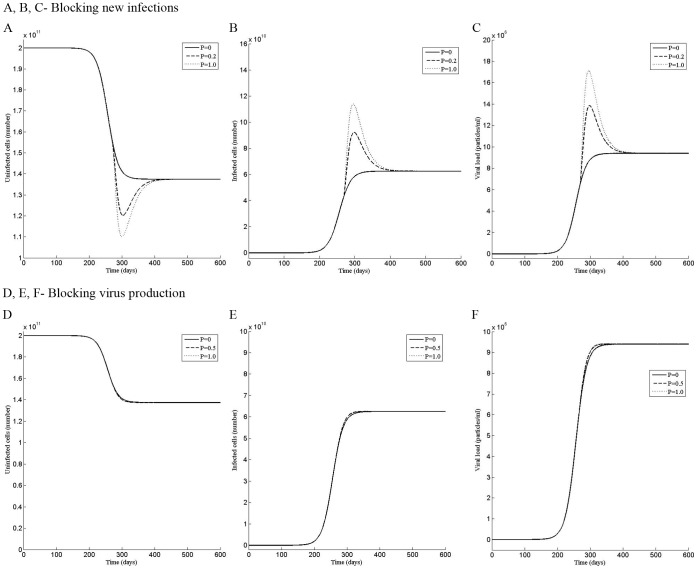
Perturbation in the third phase does not alter the steady state of the infection. Dynamics of the (A) uninfected cells, (B) infected cells, and (C) the viral load when the system was perturbed by the dose P of the drug that blocks new infections during the time period t = 270 to t = 450 day. Dynamics of the (D) uninfected cells, (E) infected cells, and (F) the viral load when the system was perturbed by the dose P of the drug that blocks virus production during the time period t = 270 to t = 450 day.

## Results

### Sensitivity Analysis of the Model Shows Different Phases of Effectiveness of the Drug

First, we performed the sensitivity analysis on the standard model with respect to the two efficacy parameters. Interestingly, since the slope of the sensitivity coefficient with time varies (Fig. 1 A, B, C), the analysis shows that there are four phases in which the response of the infection to the drug treatment may differ.

For the infected cells and the viral load, the magnitude of the sensitivity coefficient in the first phase (0≤t<165) is lower than that in the second phase (165≤t<260) while the slopes of the sensitivity curve in both phases are negative (Fig. 1 B, C, Fig. S1 B, C, E, F and Fig. S2 B, C, E, F), suggesting that a perturbation P applied in these phases will decrease the number of infected cells and the viral load with time (equation 11 and 15) relative to those without perturbation. In contrast, in the third phase (260≤t<500), the slope of the sensitivity curve is positive, suggesting that the infected cells and the viral load will increase with time if a perturbation is applied in this phase. In the fourth phase (t≥500), the slope of the sensitivity coefficient vs time plot is zero, suggesting that a perturbation may not affect the infection in this phase.

Similar to the viral load, four phases are also observed in sensitivity plots of the uninfected cells (Fig. 1 A and fig. S1 A, D; fig. S2 A, D). In contrast to the viral load, for the uninfected cells, the slope of the sensitivity coefficient is positive in the first and second phases and negative in the third phase. Since an increase in the viral load will decrease the number of uninfected cells, an effect on the viral load will appear in the opposite manner on the uninfected cells. Although the magnitudes are different, the sensitivity plot of the viral load is similar to that of the infected cells, as expected since the viral particles are produced by the infected cells.

Interestingly, the slopes of the sensitivity coefficients of the uninfected and infected cells with respect to the two efficacy parameters are similar in all phases, suggesting that the response of the system to the perturbations carried out with respect to the two modes of the drug may also be similar.

### Perturbation in the First and Second Phases Shows Two Steady States of the Infection

After observing that there are multiple phases in the sensitivity of the infection, we perturbed the model for 180 days to simulate the period of drug treatment by using increasingly higher values of the dose P of the drug that blocks new infections or virus production. For the first phase, the model was perturbed for the period t = 0 to t = 180 day and for the second phase from t = 80 to t = 260 day.

At the beginning of the infection, the total number of hepatocytes is taken 2×10^11^ [20] and infected hepatocytes are zero. As the infection develops, more and more uninfected cells become infected, increasing the viral load. With time, the rate of conversion of uninfected cells to infected cells first increases, reaching a point of inflection, then decreases to zero, reaching the steady state (Fig. 2 A, B, D, E and Fig. 3 A, B, D, E). Concurrently, the viral load increases at increasingly rapid rate till the point of inflection, then, slows to reach the steady state (Fig. 2 C, F and Fig. 3 C, F). Without perturbation (P = 0), the model reaches a steady state, having a viral load 9.4×10^6^, 1.375×10^11^ uninfected and 6.25×10^10^ infected cells, representing a state in which 31.25% of hepatocytes are infected with the HCV virus. The unperturbed steady state viral load of 9.4×10^6^ has been chosen for illustration, which is the mean initial viral load reported by Neumann et al. [20] from the patients’ data.

For the drug that blocks new infections, a perturbation of P = 0.003 in the first phase, slows the approach to the steady state by delaying the point of inflection. However, it does not alter the steady state and the uninfected, infected cells and the viral load reach the same steady state as the unperturbed model (Fig. 2 A, B, C). In contrast a stronger perturbation of P = 0.005 causes the infected cells and the viral load to decrease to zero and remain zero even after the perturbation has been withdrawn at t = 180 days (Fig. 2 B, C), altering the steady state to an uninfected state. A similar dynamics is observed in the second phase, in which, a perturbation of P = 0.003, does not affect the steady state while a perturbation of P = 0.004, permanently alters it to the uninfected state (Fig. 3 A, B, C).

The response to the perturbation by the drug that blocks virus production (Fig. 2 D, E, F and Fig. 3 D, E, F) was similar to that by the drug that blocks new infections (Fig. 2 A, B, C and Fig. 3 A, B, C), except higher strength of the perturbation was needed to alter the dynamics, suggesting that the drug, which blocks new infections, may be more potent than that, which blocks the virus production.

**Figure 5:**
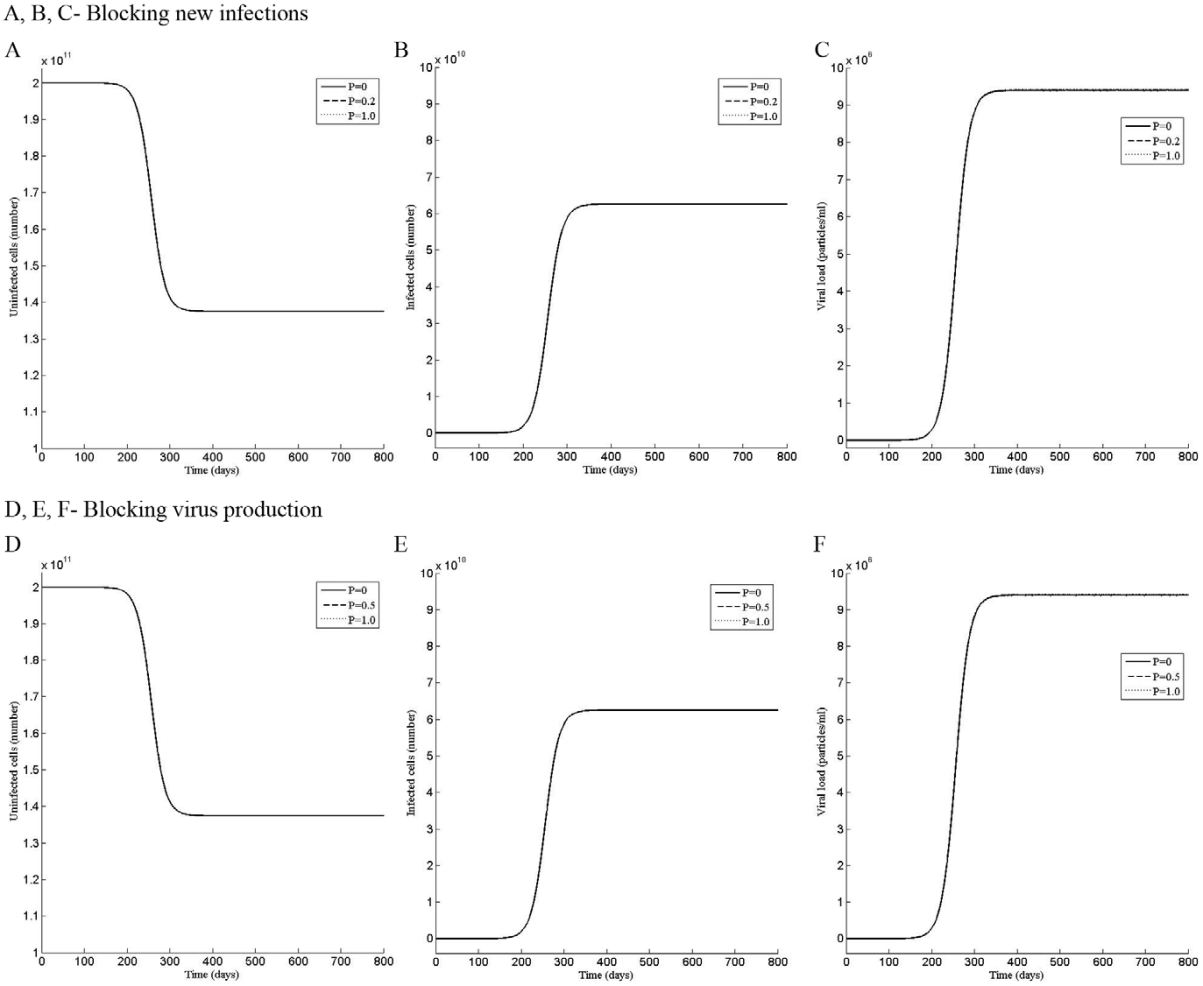
Perturbation in the fourth phase does not alter the steady state of the infection. Dynamics of the (A) uninfected cells, (B) infected cells, and (C) the viral load when the system was perturbed by the dose P of the drug that blocks new infections during the time period t = 560 to t = 740 day. Dynamics of the (D) uninfected cells, (E) infected cells, and (F) the viral load when the system was perturbed by the dose P of the drug that blocks virus production during the time period t = 560 to t = 740 day.

### The Perturbation in the Third or Fourth Phase does not Alter the Steady State of the Infection

To examine the effect of drug therapy in the third (260≤t<500) or fourth phase (t>500), the model was perturbed for 6 months from t = 270 to t = 450 day or from t = 560 to t = 740 day. Interestingly, in the third phase, although an increase in the perturbation strength decreases the number of uninfected cells and increases the infected cells and the viral load, the steady state remains unaffected (Fig. 4 A, B, C). If the mode of action of the drug is to block virus production, the perturbation has no significant effect on the infection in either of the phases (Fig. 4 D, E, F, and 5 D, E, F). Similarly, perturbation by the drug that blocks *de novo* infections does not affect the infection in the last phase (Fig. 5 A, B, C), suggesting that it will be difficult to alter the infected state in the late phases.

**Figure 6:**
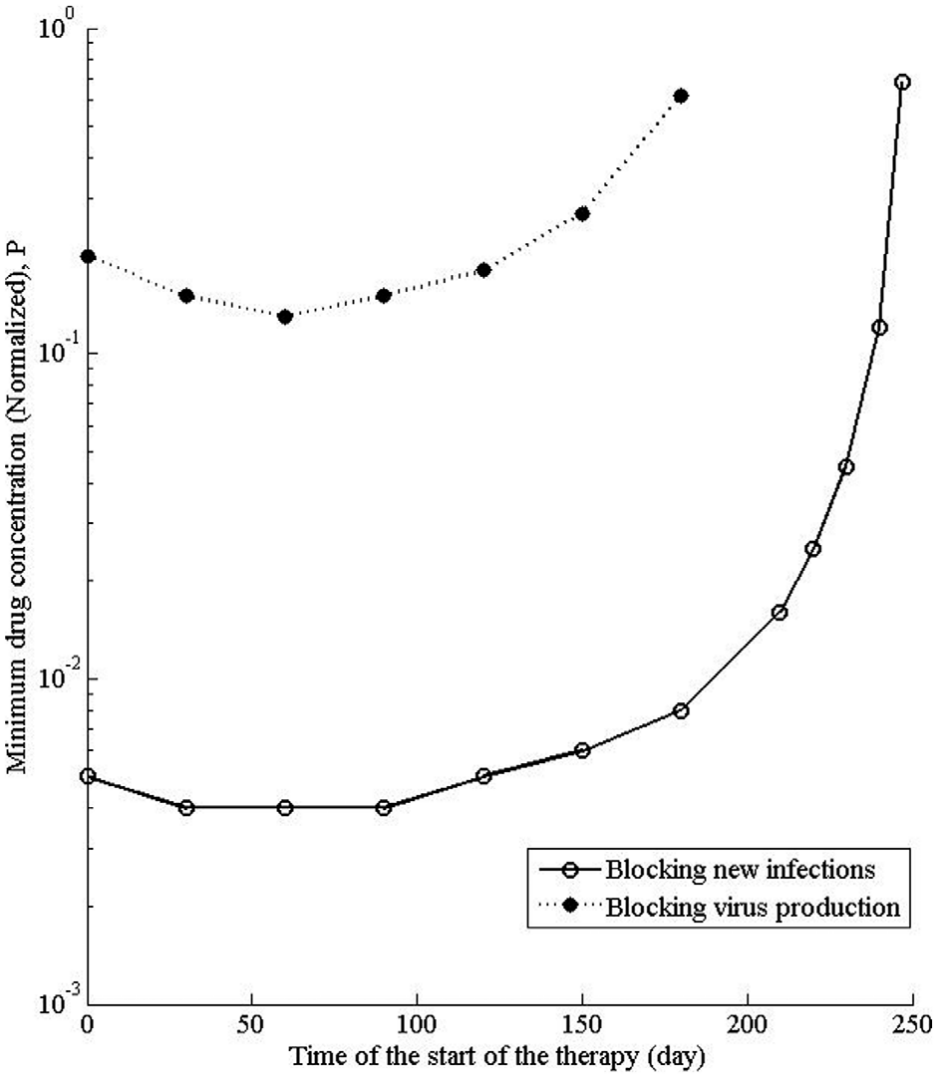
Effect of the time of start of the therapy on the state of the infection. The model has been perturbed for 180 days, starting at a specific day (x-axis) after the start of the infection. The minimum value of the normalized dose P, required to alter the steady state from “infected” to “uninfected” has been determined and plotted.

### Effect of the Time of Start of the Therapy on the State of the Infection

Since the treatment in different phases has different effect on the outcome, we determined the minimum concentration of the drug needed to alter the steady state if the therapy is carried out for 180 days, starting at different times from the start of the infection. Interestingly, for both modes of action of the drug, there is a minima (Fig. 6), corresponding to the lowest dose that could alter the steady state, suggesting that there is an optimum time to start the therapy. For the drug that blocks *de novo* infections, the minima occurs if the therapy has been started within 30–90 days. Similarly for the drug that blocks virus production, the minima occurs if the therapy has been started at around 60 day from the start of the infection (Fig. 6). As the therapy duration, starting in the second phase, expands into the third phase, the minimum dose of the drug required to alter the steady state increases rapidly (Fig. 6), suggesting that clearing the infection may become progressively difficult with the delay in the start of the therapy.

## Discussion

Interestingly, the sensitivity analysis of the virus dynamics model with respect to the two efficacy parameters showed multiple phases, suggesting that the time of the start of the therapy may be an important factor in determining the response. The magnitude of the sensitivity coefficient describes how the uninfected, infected cells and the viral load will change at a given time if the efficacy of the drug is varied at that time. On the other hand, the slope of the sensitivity coefficient vs time plot describes how the uninfected, infected cells and the viral load will vary with time if a drug of a fixed efficacy is applied during a time period (equation 11 and 15).

If a 6 month therapy is carried out in the first or the second phase of the infection, the increase in the dose of the drug steadily delays the point of inflection in the dynamics, delaying the approach to the steady state. As the dose is further increased to a certain value, the uninfected, infected cells and the viral load do not reach the same steady state as they did for the lower doses but the infected cells and the viral load decrease to zero while the uninfected cells increase to the same number as the total number of the cells. Thus, the steady state of the infection has been altered from a 31.25% infected cells and 9.4×10^6^ viral load to an uninfected state. It also suggests that a critical efficacy of the drug is needed to achieve the SVR as described by others [26], [27]. For the same mode of the drug, in the first and second phases, the doses required to achieve the SVR are nearly the same. In addition, in these phases, the alteration of the steady state is very sensitive to the dose and a small increase in the perturbation could change the steady state from the “infected state” to an “uninfected state”. These effects are likely due to the early phase of the infection. As the viral load and infected cells decline, the number of uninfected cells increases. When the viral load reaches zero during the therapy, there are no virus particles left to propagate the infection and the cells remain uninfected even after the therapy has been withdrawn. A lower dose of the drug may be needed to achieve SVR if the mode of action of the drug is to block the *de novo* infections than to block virus production since the former affects the new infections directly while the later affects it indirectly by reducing the plasma virus concentration.

In contrast, in the third phase, due to the positive slope of the sensitivity curve for the viral load and infected cells, the drug serves as an activator of the infection (equation 11 and 15). It causes the viral load and the infected cells to increase. The slopes of the sensitivity curves in this phase gradually changes to zero (Fig. 1 B, C) so is the effect of the drug. When the perturbation is withdrawn, the system returns to the infected state.

In the final phase, the infection has reached the steady state in which the uninfected, infected cells and the viral load have attained equilibrium. Thus, all the sensitivity coefficients have attained constant values (Fig. 1 A, B, C). Since, the slope of the sensitivity coefficient vs time plot is zero in this phase, a drug may not perturb the system either as an activator or a repressor.

Time of start of the therapy is an important factor in determining the outcome. For both modes of action of drugs, there is an optimum time to start the treatment. Since the magnitude of the slope of the sensitivity curve for the infected cells and the viral load progressively increases in the first and second phases, reaching a point of inflection in the second phase (Fig. S2 B, C), a therapy close to this time will be the most effective, giving the lowest dose that could alter the steady state of the infection.

Our analysis shows that blocking new infections is more effective than blocking virus production. Although significant changes in the model will be needed for it to be applicable to direct acting antivirals [41], its implications to DAAs are interesting. It can be inferred that blocking wild type virus at an early phase of HCV lifecycle is better yet the direct acting antivirals produce drug resistant viral quasispecies, depending on the phase they block. Genetic barrier to resistance of NS3/4A protease inhibitors, which block an early phase of HCV lifecycle, has been shown to be low [42], [43]. On the other hand, genetic barrier to resistance of nucleoside analog polymerase inhibitors, which block a later phase of the viral lifecycle, have been shown to be high while that of the nonnucleoside polymerase inhibitor is low [42], [43]. Therefore, there may be optimum phases at which a drug may be highly effective in terms of both blocking the wild type infection and limiting the number of drug resistant viral quasispecies.

From the present study, we conclude that the treatment during the first and second phases of the infection will likely result in SVR, explaining the recent clinical studies [44]–[48]. Therefore, development of better HCV screening tools is needed so that the infection is detected early on. Furthermore, we found that the drug that blocks *de novo* infections is more effective in achieving the SVR.

## Supporting Information

Figure S1 **Variation of the sensitivity coefficient in the first phase of the infection.** Sensitivity coefficient of the (A) uninfected cells, (B) infected cells, and (C) the viral load with respect to the efficacy ε_n_ was plotted for the time period t = 0 to t = 165 day for ε_p_ = 0 and ε_n_ = 0. Sensitivity coefficient of the (D) uninfected cells, (E) infected cells, and (F) the viral load with respect to the efficacy ε_p_ was plotted for the time period t = 0 to t = 165 day for ε_p_ = 0 and ε_n_ = 0.
(TIF)

Figure S2 **Variation of the sensitivity coefficient in the second phase of the infection.** Sensitivity coefficient of the (A) uninfected cells, (B) infected cells, and (C) the viral load with respect to the efficacy ε_n_ was plotted for the time period t = 165 to t = 260 day for ε_p_ = 0 and ε_n_ = 0. Sensitivity coefficient of the (D) uninfected cells, (E) infected cells, and (F) the viral load with respect to the efficacy ε_p_ was plotted for the time period t = 165 to t = 260 day for ε_p_ = 0 and ε_n_ = 0.
(TIF)
