# Supplementary figures and images for: Analysis of the Virus Dynamics Model Reveals That Early Treatment of HCV Infection May Lead to the Sustained Virological Response
Source: PLoS One. 2012 Jul 24;7(7):e41209. doi: 10.1371/journal.pone.0041209 (PMC3404063; doi:10.1371/journal.pone.0041209)

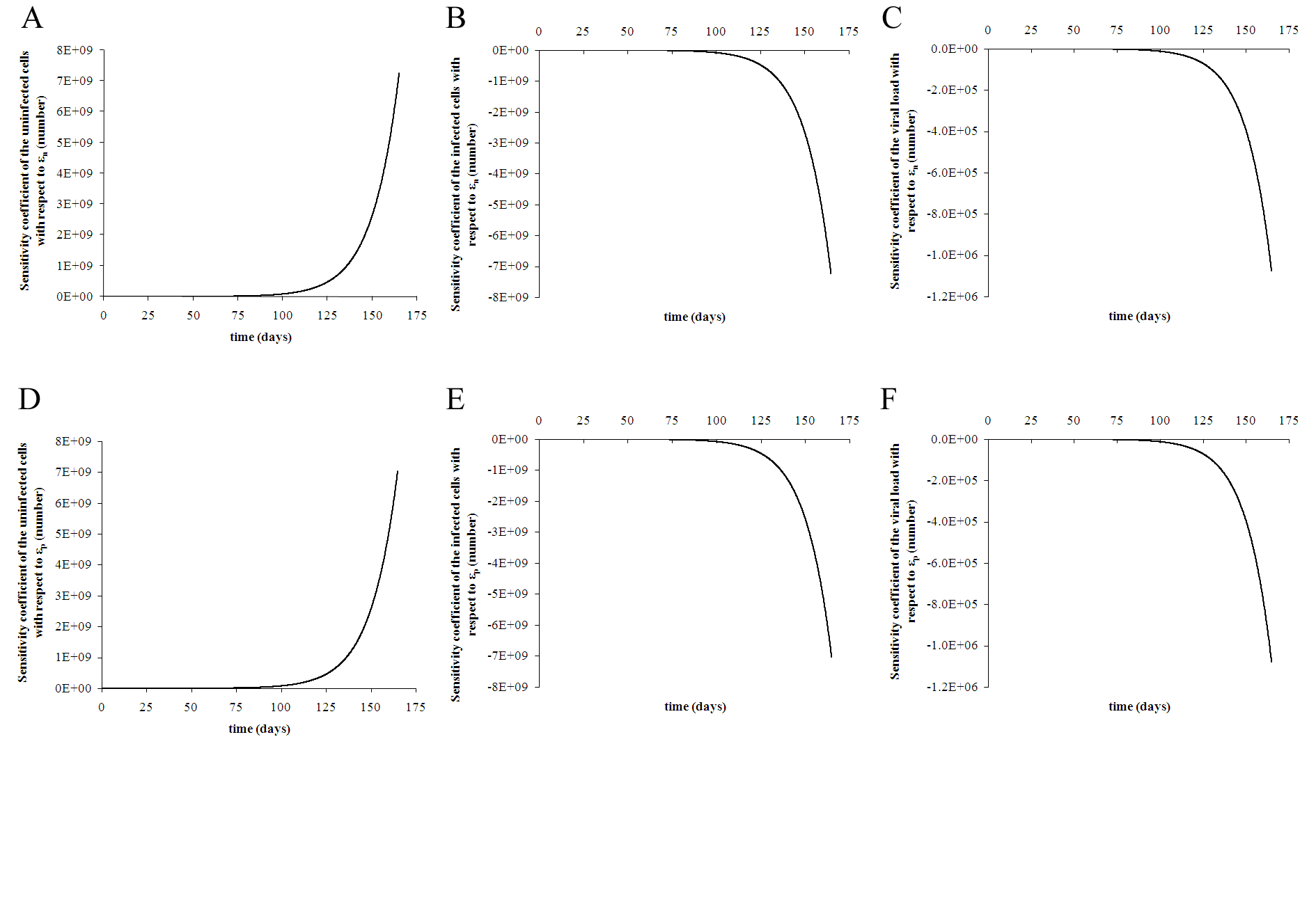

Supplement: Figure S1 — Variation of the sensitivity coefficient in the first phase of the infection. Sensitivity coefficient of the (A) uninfected cells, (B) infected cells, and (C) the viral load with respect to the efficacy εn was plotted for the time period t = 0 to t = 165 day for εp = 0 and εn = 0. Sensitivity coefficient of the (D) uninfected cells, (E) infected cells, and (F) the viral load with respect to the efficacy εp was plotted for the time period t = 0 to t = 165 day for εp = 0 and εn = 0. (TIF) [file pone.0041209.s001.tif]

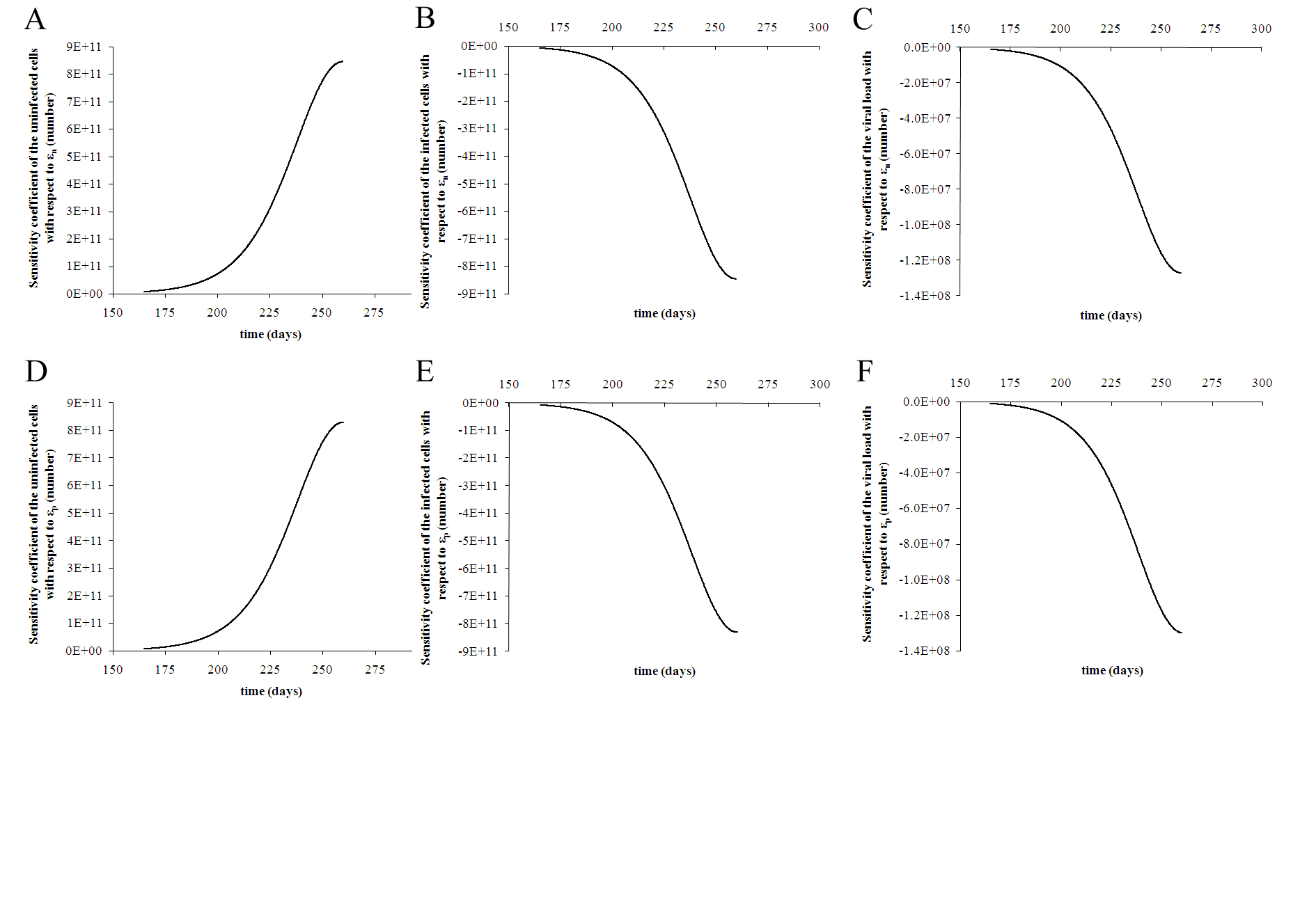

Supplement: Figure S2 — Variation of the sensitivity coefficient in the second phase of the infection. Sensitivity coefficient of the (A) uninfected cells, (B) infected cells, and (C) the viral load with respect to the efficacy εn was plotted for the time period t = 165 to t = 260 day for εp = 0 and εn = 0. Sensitivity coefficient of the (D) uninfected cells, (E) infected cells, and (F) the viral load with respect to the efficacy εp was plotted for the time period t = 165 to t = 260 day for εp = 0 and εn = 0. (TIF) [file pone.0041209.s002.tif]
